# Unfavorable social determinants of health are associated with higher burden of financial toxicity among patients with atherosclerotic cardiovascular disease in the US: findings from the National Health Interview Survey

**DOI:** 10.1186/s13690-022-00987-z

**Published:** 2022-12-06

**Authors:** Javier Valero-Elizondo, Zulqarnain Javed, Rohan Khera, Mauricio E. Tano, Ramzi Dudum, Isaac Acquah, Adnan A. Hyder, Julia Andrieni, Garima Sharma, Michael J. Blaha, Salim S. Virani, Ron Blankstein, Miguel Cainzos-Achirica, Khurram Nasir

**Affiliations:** 1 Department of Cardiology, Division of Cardiovascular Prevention and Wellness, Houston Methodist DeBakey Heart & Vascular Center, 7550 Greenbriar Drive, Houston, TX 77030 USA; 2 Center for Outcomes Research, Houston Methodist, 7550 Greenbriar Drive, Houston, TX 77030 USA; 3 Department of Internal Medicine, Section of Cardiovascular Medicine, Yale School of Medicine, New Haven, CT USA; 4 Center for Outcomes Research and Evaluation, Yale-New Haven Hospital, New Haven, CT USA; 5 Division of Cardiovascular Medicine, Stanford University, Stanford, CA USA; 6 Milken Institute School of Public Health, George Washington University, Washington, DC USA; 7 Population Health and Primary Care, Houston Methodist Hospital, Houston, TX USA; 8 Department of Medicine, Division of Cardiology, Johns Hopkins University School of Medicine, Baltimore, MD USA; 9 The Johns Hopkins Ciccarone Center for the Prevention of Cardiovascular Disease, Baltimore, MD USA; 10 Section of Cardiology, Michael E. DeBakey Veterans Affairs Medical Center, Houston, TX USA; 11 Health Policy, Quality & Informatics Program, Michael E. DeBakey VA Medical Center Health Services Research & Development Center for Innovations in Quality, Effectiveness, and Safety, Houston, TX USA; 12 Department of Medicine, Section of Cardiology, Baylor College of Medicine, Houston, TX USA; 13 Department of Medicine, Section of Cardiovascular Research, Baylor College of Medicine, Houston, TX USA; 14 Cardiovascular Imaging Program, Cardiovascular Division and Department of Radiology, Brigham and Women’s Hospital, Boston, MA USA

**Keywords:** Cardiovascular disease, Disparities, Equity, Financial toxicity, Social determinants of health

## Abstract

**Background:**

Atherosclerotic cardiovascular disease (ASCVD) is a major cause of financial toxicity, defined as excess financial strain from healthcare, in the US. Identifying factors that put patients at greatest risk can help inform more targeted and cost-effective interventions. Specific social determinants of health (SDOH) such as income are associated with a higher risk of experiencing financial toxicity from healthcare, however, the associations between more comprehensive measures of cumulative social disadvantage and financial toxicity from healthcare are poorly understood.

**Methods:**

Using the National Health Interview Survey (2013–17), we assessed patients with self-reported ASCVD. We identified 34 discrete SDOH items, across 6 domains: economic stability, education, food poverty, neighborhood conditions, social context, and health systems. To capture the cumulative effect of SDOH, an aggregate score was computed as their sum, and divided into quartiles, the highest (quartile 4) containing the most unfavorable scores. Financial toxicity included presence of: difficulty paying medical bills, and/or delayed/foregone care due to cost, and/or cost-related medication non-adherence.

**Results:**

Approximately 37% of study participants reported experiencing financial toxicity from healthcare, with a prevalence of 15% among those in SDOH Q1 vs 68% in SDOH Q4. In fully-adjusted regression analyses, individuals in the 2nd, 3rd and 4th quartiles of the aggregate SDOH score had 1.90 (95% CI 1.60, 2.26), 3.66 (95% CI 3.11, 4.35), and 8.18 (95% CI 6.83, 9.79) higher odds of reporting any financial toxicity from healthcare, when compared with participants in the 1st quartile. The associations were consistent in age-stratified analyses, and were also present in analyses restricted to non-economic SDOH domains and to 7 upstream SDOH features.

**Conclusions:**

An unfavorable SDOH profile was strongly and independently associated with subjective financial toxicity from healthcare. This analysis provides further evidence to support policies and interventions aimed at screening for prevalent financial toxicity and for high financial toxicity risk among socially vulnerable groups.

**Supplementary Information:**

The online version contains supplementary material available at 10.1186/s13690-022-00987-z.

## Introduction

Atherosclerotic cardiovascular disease (ASCVD) is a major cause of financial toxicity – defined as excess financial strain from healthcare – in the US [1]. The burden of financial toxicity is particularly high among non-elderly adults [1, 2]. Patients with ASCVD experience higher medical expenditures than those without [3], a concerning 50% of ASCVD patients younger than 65 years report having difficulty paying their medical bills, and 20% are unable to pay them at all [4]. Further, those with ASCVD in the US report a significant prevalence of cost-related medication non-adherence [5], and frequent foregone/delayed care due to cost [6].

Although efforts to prevent and mitigate financial toxicity from healthcare should be pursued in all patients with ASCVD, identifying factors that put patients at greatest risk can help inform more targeted and cost-effective interventions. In this context, specific social determinants of health (SDOH) such as low income have been shown to be independent predictors of financial toxicity, particularly in adults aged < 65 years. Along the same lines, low income is independently associated with higher out-of-pocket health-related expenditures and catastrophic expenditures [7], as well as higher overall health-related financial burden in patients with ASCVD [8].

Beyond socioeconomic position, several frameworks have identified additional important dimensions of SDOH, inclusive of neighborhood environment, community and social context, food poverty, education and access to healthcare [9, 10]. However, in the context of this broader framework, the associations between a higher cumulative social disadvantage and the burden of financial toxicity from healthcare are poorly understood among US patients with ASCVD. To the best of our knowledge, no prior large-scale studies in the US have used a comprehensive SDOH framework to capture the extent of social disadvantage in patients with ASCVD, and examine its association with financial toxicity. The aims of this study were thus to 1) fill this knowledge gap using large-scale, population-based, nationally representative data, and 2) assess whether these associations vary across age strata. We tested the hypothesis that higher cumulative social disadvantage is associated with higher burden of financial toxicity from healthcare.

## Methods

### Data source and study design

The National Health Interview Survey (NHIS), compiled by the National Center for Health Statistics/Center for Disease Control and Prevention, is a series of cross-sectional national surveys conducted annually. Complex, multi-stage sampling methods are used so that NHIS generates estimates that are generalizable to the noninstitutionalized US population [11]. The questionnaires collect a breadth of information at the household, family, and personal levels, including but not limited to: sociodemographic characteristics, indicators of health status, activity limitations, injuries, health insurance coverage, access to and utilization of health care services, and health-related behaviors on the US population [12].

For the present cross-sectional analysis, we pooled NHIS data for years 2013 to 2017. Pooling NHIS data for several survey years is common practice, and helps maximize power, reliability, and generalizability of the findings [13]. The study period was restricted to years 2013 to 2017 due to some SDOH variables being absent before 2013 or starting 2018. Given the public availability and de-identified nature of the NHIS data, this study was exempt from the purview of Houston Methodist Hospital’s Institutional Review Board Committee [14]. No extramural funding was used to support this work.

### Study population

We included all adult (aged ≥18 years) NHIS participants who reported a history of ASCVD, inclusive of angina, myocardial infarction, other coronary heart disease, and stroke. Specifically, individuals were considered to have prevalent ASCVD if they responded “Yes” to at least one of the following 4 questions: “Have you ever been told by a doctor or other health professional that you had … coronary heart disease?”, “… angina, also called angina pectoris?”, “… a heart attack (also called myocardial infarction)?”, “have you ever been told by a doctor or other health professional that you had a stroke?” [4, 15].

Prior studies in the US suggest that the phenomenon of financial toxicity from healthcare is more frequent among individuals aged < 65 years who have chronic diseases, while this phenomenon is attenuated at age 65, when Medicare and other social benefits become available [16]. Consequently, we defined two main subgroups based on age at the time of participation in NHIS: non-elderly (< 65 years of age) and elderly (≥65 years of age) participants.

### Aggregate SDOH score

We used the SDOH framework described by the Kaiser Family Foundation (KFF) [9], which includes 6 domains: 1) economic stability, 2) neighborhood, 3) community and social context, 4) food poverty, 5) education, and 6) access to healthcare. Specifically, and using the vast SDOH information collected in NHIS, we constructed a list of 34 individual components across those six domains (Supplemental Table 1). Each component was collected in NHIS, and for the purposes of the present analysis each of them was classified as either favorable or unfavorable, being assigned a value of 0 for the former and of 1 for the latter, respectively (e.g., insured = 0 vs. uninsured = 1; middle/high-income = 0 vs. low-income = 1).

We then created an SDOH aggregate score by combining all 34 components. The distribution of the SDOH aggregate score in the study population was divided into quartiles, with the most favorable (i.e. lowest) SDOH scores in the 1st quartile and the most unfavorable (i.e. highest) SDOH scores in the 4th quartile.

### Subjective financial toxicity from healthcare

For the present analysis we were interested in three manifestations of subjective financial toxicity from healthcare, as reported by study participants: difficulty paying medical bills, cost-related medication non-adherence, and/or delayed/foregone care due to cost.

The following questions were used in NHIS to assess difficulty paying medical bills: “In the past 12 months, did you/anyone in your family have problems paying or were unable to pay any medical bills? Include bills for doctors, dentists, hospitals, therapists, medication, equipment, nursing home or home care”, and/or “do you/anyone in your family currently have any medical bills that are being paid off over time? This could include medical bills being paid off with a credit card, through personal loans, or bill paying arrangements with hospitals or other providers. The bills can be from earlier years as well as this year”. This approach has been previously employed in other studies and surveys [17, 18]. For individuals who answered “Yes”, a follow-up question was asked: “Do you/Does anyone in your family currently have any medical bills that you are unable to pay at all?”. These questions defined 4 scenarios with regards to difficulty paying medical bills: no, any, difficulty paying medical bills but able to pay, inability to pay medical bills at all; the last two being mutually exclusive. Cost-related medication non-adherence was defined as a participants reporting any of the following behaviors in order to save money in the last 12 months: skipping medication doses, taking less medicine or delaying filling a prescription [5]. Delayed and/or foregone care due to cost was assessed by asking participants whether, within the past year, medical care had been delayed due to cost, or if they needed but did not received medical care due to cost (Supplemental Table 2) [19].

Using this information, we defined 1) “any financial toxicity”, as presence of at least one of the following: any difficulty paying medical bills, cost-related medication non-adherence, delayed/foregone care due to cost; and 2) an ordinal variable for number of components, categorized as 0, 1, and ≥ 2.

### Other covariates

Other variables used in this analysis included sex, race/ethnicity, region, family income, cardiovascular risk factors, and chronic comorbidities. All were self-reported. Race/ethnicity included non-Hispanic White, non-Hispanic Black, non-Hispanic Black, and Hispanic. Region was reported using the US Census Bureau’s four statistical regions: Northeast, Midwest, South and West. Family income was based on percent of family income to the federal poverty level from the Census Bureau: middle/high-income (≥200%), and low-income (< 200%). Cardiovascular risk factors included hypertension, diabetes mellitus, high cholesterol, obesity (calculated body mass index ≥30 kg/m^2^), current smoker, and insufficient physical activity (based on not participating in > 150 minutes per week of moderate-intensity aerobic physical activity, > 75 minutes per week of vigorous-intensity aerobic physical activity, or a total combination of ≥150 minutes per week of moderate/vigorous-intensity aerobic physical activity). Self-reported chronic comorbidities included: emphysema, chronic obstructive pulmonary disease, asthma, gastrointestinal ulcer, cancer (any), musculoskeletal conditions (including arthritis, gout, fibromyalgia, rheumatoid arthritis, and systemic lupus erythematosus), liver disease and kidney disease. The number of these comorbidities was also quantified and categorized as 0, 1 or ≥ 2.

### Statistical analysis

We used survey weighted proportions to describe the characteristics of study participants, overall and by age strata, as well as the burden of financial toxicity (overall, of its individual components, and number of components). We also described the prevalence of any financial toxicity and the distribution of number of components further stratifying by SDOH quartiles.

We used logistic regression models to estimate the associations between higher SDOH quartiles (using the 1st as reference) and prevalence of any financial toxicity, overall and among age strata. To adjust for potential confounders, we constructed 3 sequential models - our base model (model 1) adjusted only for age and sex; model 2 also adjusted for race/ethnicity; and model 3 further adjusted for cardiovascular risk factors and comorbidities. Interaction by age was tested by adding an interaction term to the fully adjusted model (model 3).

Similarly, we used polynomial regression to assess the associations between higher SDOH quartiles and number of financial toxicity components (using 0 components as reference), overall and among age strata. The same multivariable models and interaction tests described above were conducted for this analysis.

Income is a powerful independent predictor of financial hardship from medical bills [8], therefore, to evaluate the relationship between non-income cumulative SDOH disadvantage and financial toxicity, in a sensitivity analysis we further stratified our descriptive analyses of financial toxicity prevalence by family income (middle/high-income, and low-income), and removed income from the SDOH aggregate score (which now had 33 components rather than 34). Second, since some factors in our aggregate SDOH score could be considered financial toxicity themselves (e.g., financial distress, food insecurity), we conducted an additional sensitivity analysis using only 7 mostly upstream SDOH factors: family income, house tenure, English language proficiency, education attainment, employment, insurance status, and usual source of care. Similarly to the approach used in the main analysis, these 7 variables were each scored 0–1, summed, and the resulting distribution was categorized as 0, 1, 2, or ≥ 3 (~ 25% prevalence each). We also examined the association between each of these 7 individual SDOH and financial toxicity. Further, we tested for possible variation in the effects of “monetary” (i.e. *economic stability* domain) vs “non-monetary” (*neighborhood, food poverty, community and social context, education* and *access to healthcare* domains) SDOH factors on financial toxicity, using separate logistic regression models.

Finally, to assess the importance of different SDOH domains in the association between cumulative SDOH disadvantage and financial toxicity, we used a random forest model to determine the percentage of attributable risk to each one of the six major SDOH domains.

Person-level weights are created by NHIS based on the probability of selection for each participant, adjusted for non-response, and further adjusted for poststratification by age, sex and race/ethnicity classes based on population estimates produced by the US Census Bureau. Variance estimation for the entire pooled cohort was obtained from the Integrated Public Use Microdata Series (http://www.ipums.org) [20], and confidence intervals were calculated through Stata’s “svy” command – used for all analyses – which automatically accounts for the complex survey design of the survey, as per the NHIS’ recommendations [11].

For all analyses, a *p* value < 0.05 was considered statistically significant. All analyses were carried out using Stata version 16 (StataCorp, LP, College Station, Texas, USA).

## Results

### Study population

Between years 2013 and 2017, the NHIS included 164,696 participants ≥18 years of age. Of them, 15,758 reported a history of ASCVD (weighted prevalence: 8.1%), which translates to 19.6 million US adults annually. Those 15,758 NHIS participants with prevalent ASCVD defined the study population for the present analysis. Mean age was 65.3 years (standard deviation 15.5). There was a high proportion of men, and a high burden of cardiovascular risk factors and other comorbidities (Table 1).

**Table 1:** Characteristics of adult National Health Interview Survey participants with atherosclerotic cardiovascular disease, years 2013 to 2017

Characteristics	Overall (***N*** = 15,758) % (95% CI)	< 65 (***N*** = 6160) % (95% CI)	≥65 (***N*** = 9598) % (95% CI)
Age, mean (standard deviation)	65.3 (15.5)	52.4 (10.4)	75.5 (7.6)
Age strata			
18–44	8.5 (7.7, 9.2)	–	–
45–64	35.8 (34.7, 36.9)	–	–
≥ 65	55.7 (54.6, 56.8)	–	–
Sex			
Male	56.4 (55.4, 57.4)	57.5 (55.9, 59.2)	55.5 (54.2, 56.8)
Female	43.6 (42.6, 44.6)	42.5 (40.8, 44.1)	44.5 (43.2, 45.8)
Race/Ethnicity			
Non-Hispanic White	74.6 (73.4, 75.8)	67.1 (65.3, 68.9)	80.6 (79.3, 81.7)
Non-Hispanic Black	12.2 (11.4, 13.1)	16.4 (15.1, 17.7)	9.0 (8.2, 9.8)
Non-Hispanic Asian	3.0 (2.6, 3.4)	2.8 (2.3, 3.5)	3.1 (2.7, 3.7)
Hispanic	10.1 (9.3, 11.1)	13.7 (12.3, 15.2)	7.3 (6.5, 8.2)
Family Income			
Middle/High-income	59.1 (57.9, 60.2)	53.8 (52.1, 55.4)	63.5 (62.1, 64.9)
Low-income	40.9 (39.8, 42.1)	46.2 (44.5, 47.9)	36.5 (35.0, 37.9)
Region			
Northeast	16.9 (15.9, 18.0)	15.2 (13.9, 16.7)	18.3 (17.0, 19.6)
Midwest	24.7 (23.5, 25.9)	24.4 (22.7, 26.1)	25.0 (23.5, 26.4)
South	40.0 (38.6, 41.4)	42.4 (40.1, 44.3)	38.0 (36.4, 39.7)
West	18.4 (17.3, 19.6)	18.0 (16.7, 19.5)	18.7 (17.4, 20.2)
Hypertension	72.8 (71.8, 73.8)	67.3 (65.6, 68.9)	77.3 (76.1, 78.3)
High cholesterol	63.5 (62.5, 64.5)	58.7 (57.0, 60.4)	67.4 (66.2, 68.6)
Active smoking	18.5 (17.6, 19.3)	28.9 (27.4, 30.5)	10.1 (9.4, 10.9)
Obesity	39.6 (38.6, 40.7)	47.6 (45.9, 49.4)	33.3 (32.1, 34.5)
Insufficient physical activity	68.5 (67.5, 69.6)	64.7 (63.0, 66.4)	71.6 (70.4, 72.7)
Comorbidities			
0	26.1 (25.2, 27.1)	32.7 (31.1, 34.2)	21.0 (19.9, 22.0)
1	33.1 (32.1, 34.0)	31.0 (29.4, 32.6)	34.8 (33.6, 36.0)
≥ 2	40.8 (39.7, 41.8)	36.4 (34.7, 38.0)	44.3 (42.9, 45.6)

Results are presented as number (weighted %) unless specified otherwise

Of them, 6160 were non-elderly, and 9598 were elderly, translating to 8.7 and 10.9 million US adults annually. Non-elderly individuals were more likely to be Hispanics or Non-Hispanic Blacks, from low-income households, and active smokers. In contrast, elderly individuals had a higher prevalence of cardiovascular risk factors and comorbidity count.

### Burden of financial toxicity from healthcare

The prevalence of any difficulty paying medical bills 31.0% (95% CI 30.0, 32.1), including 19.1% (95% CI 18.3, 20.0) who had difficulty paying bills but were able to pay, 11.9% (95% CI 11.2, 12.6) unable to pay their medical bills at all, 12.3% (95% CI 11.7, 13.0) who had delayed/foregone care due to cost, and 11.4% (95% CI 10.8, 12.1) reporting cost-related medication non-adherence (Table 2). The prevalence of any financial toxicity from healthcare was 36.9% (95% CI 35.8, 38.0), with 18.3% (95% CI 17.4, 19.2) having reported having one financial toxicity component, and 18.6% (95% CI 17.7, 19.5) having reported two or more. The burden of financial toxicity was more prevalent in the non-elderly when compared to the elderly across the board, including any difficulty paying medical bills (45.1% vs 19.9%), inability to pay bills at all (18.9% vs 6.3%), and any financial toxicity (53.1% vs 24.0%), respectively.

**Table 2:** Financial toxicity from healthcare among adult National Health Interview Survey participants with atherosclerotic cardiovascular disease, years 2013 to 2017

Characteristics	Overall (***N*** = 15,758)	< 65 (***N*** = 6160)	≥65 (***N*** = 9598)
Financial hardship from medical bills			
Any	31.0 (30.0, 32.1)	45.1 (43.4, 46.7)	19.9 (18.8, 21.0)
Difficulty paying medical bills but able to pay	19.1 (18.3, 20.0)	26.2 (24.7, 27.7)	13.5 (12.6, 14.5)
Unable to pay bills at all	11.9 (11.2, 12.6)	18.9 (17.6, 20.2)	6.3 (5.7, 7.1)
Delayed/foregone care due to cost	12.3 (11.7, 13.0)	(20.9 (19.7, 22.2)	5.5 (5.0, 6.1)
Cost-related medication non-adherence	11.4 (10.8, 12.1)	18.5 (17.3, 19.9)	5.8 (5.2, 6.4)
Any financial toxicity	36.9 (35.8, 38.0)	53.1 (51.4, 54.8)	24.0 (22.8, 25.2)
Number of financial toxicity components			
0	63.1 (62.0, 64.2)	46.9 (45.2, 48.6)	76.0 (74.8, 77.2)
1	18.3 (17.4, 19.2)	23.2 (21.8, 24.6)	14.4 (13.5, 15.4)
≥ 2	18.6 (17.7, 19.5)	29.9 (28.4, 31.4)	9.6 (8.8, 10.4)

Results are presented as number (weighted %)

### Interplay between SDOH and financial toxicity

**Fig. 1:**
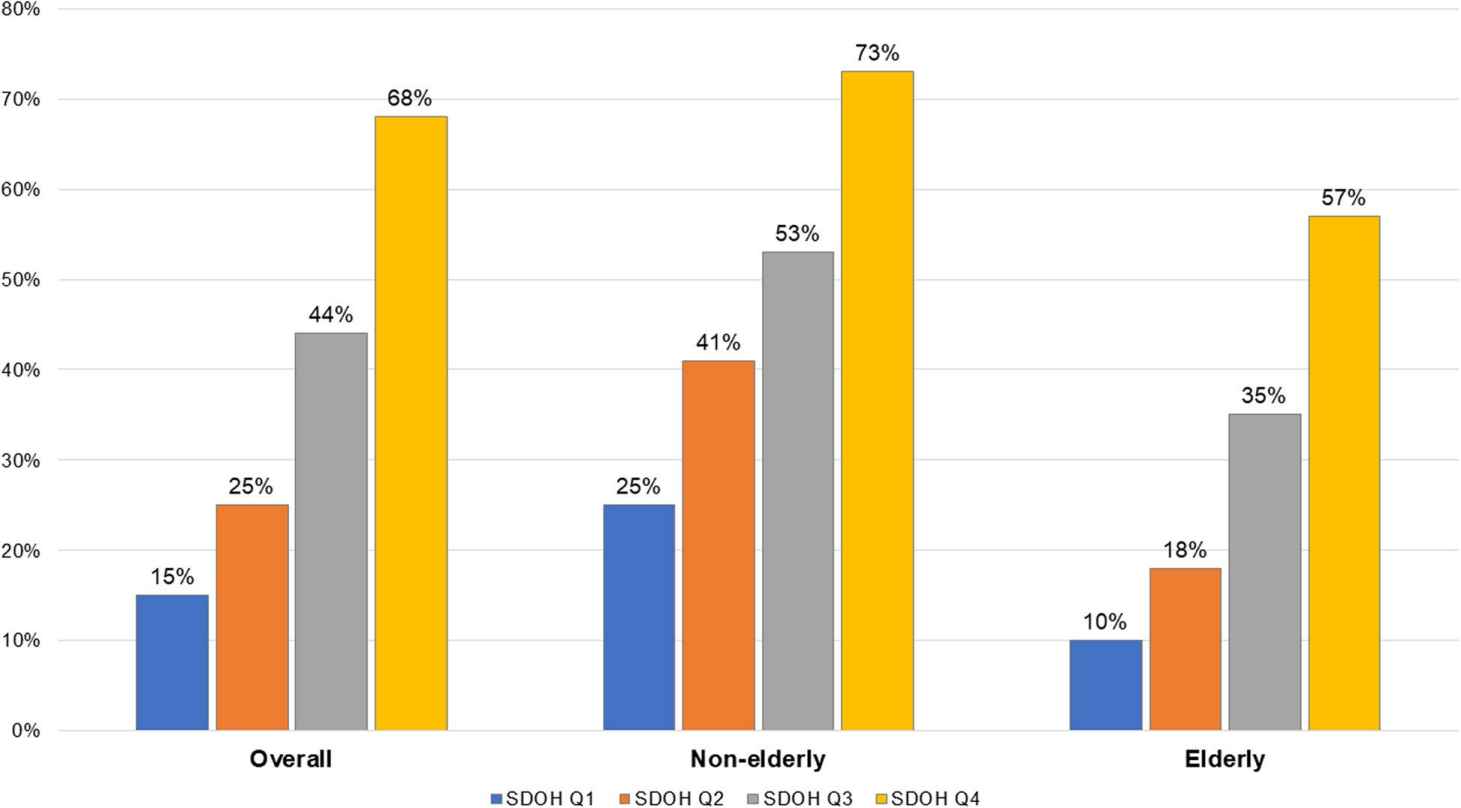
Prevalence of any financial toxicity by age and SDOH quartile, adult participants with ASCVD in the National Health Interview Survey 2013–2017. Abbreviations: ASCD, atherosclerotic cardiovascular disease; SDOH, social determinants of health

In unadjusted analyses, the higher the SDOH score quartile, the higher the prevalence of any financial toxicity from healthcare (Fig. 1). This trend was observed overall as well as among both age strata, although for each SDOH quartile, the prevalence of any financial toxicity was consistently higher in non-elderly than in elderly participants. For instance, for SDOH quartile 1, the prevalence of any financial toxicity from healthcare was 25% for non-elderly participants vs 10% in elderly participants; for SDOH quartile 4, the prevalence was 73% vs 57%, respectively.

**Fig. 2:**
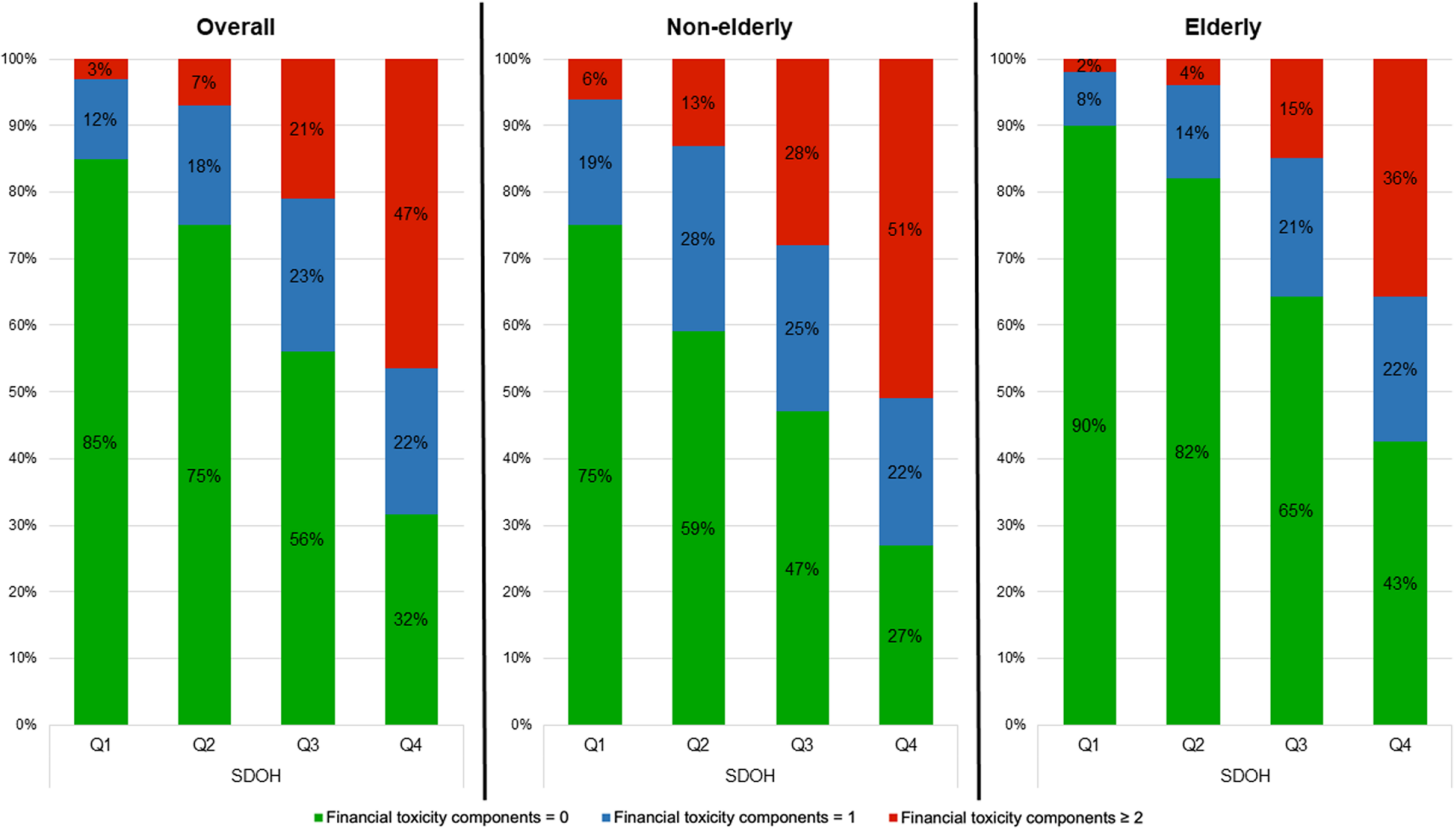
Number of financial toxicity components by age and SDOH quartile, adult participants with ASCVD in the National Health Interview Survey 2013–2017. Abbreviations: ASCD, atherosclerotic cardiovascular disease; SDOH, social determinants of health

Similar trends were observed in unadjusted analyses using number of financial toxicity components as the outcome of interest (Fig. 2).

### Multivariable associations between SDOH and financial toxicity

Adjusting for relevant covariates, higher SDOH quartiles were independently associated with higher odds of any financial toxicity from healthcare relative to lowest SDOH quartile (Table 3). In the fully adjusted model (model 3), participants in the 2nd, 3rd and 4th quartile had 1.90 (95% CI 1.60, 2.26), 3.66 (95% CI 3.11, 4.35), and 8.18 (95% CI 6.83, 9.79) higher adjusted odds of reporting any financial toxicity from healthcare compared with participants in the 1st quartile. Consistent qualitative trends were observed in analyses stratified by age, although the *p*-value for interaction was 0.003 in the context of a very large sample size.

**Table 3:** Associations between social determinants of health quartiles and any financial toxicity

	SDOH Aggregate Quartiles	Model 1^a^	Model 2^b^	Model 3^c^
**Overall**	*1st (Reference)*	*1.00*	*1.00*	*1.00*
	2nd	1.97 (1.68, 2.31)	2.01 (1.71, 2.36)	1.90 (1.60, 2.26)
	3rd	4.04 (3.47, 4.69)	4.19 (3.60, 4.89)	3.66 (3.11, 4.31)
	4th	9.24 (7.85, 10.88)	9.96 (8.42, 11.79)	8.18 (6.83, 9.79)
**< 65**	*1st (Reference)*	*1.00*	*1.00*	*1.00*
	2nd	2.08 (1.61, 2.67)	2.15 (1.67, 2.78)	2.19 (1.68, 2.86)
	3rd	3.36 (2.67, 4.21)	3.61 (2.87, 4.56)	3.55 (2.78, 4.54)
	4th	7.82 (6.22, 9.84)	8.82 (6.94, 11.21)	8.38 (6.44, 10.91)
**≥65**	*1st (Reference)*	*1.00*	*1.00*	*1.00*
	2nd	1.91 (1.56, 2.35)	1.92 (1.55, 2.36)	1.72 (1.38, 2.14)
	3rd	4.74 (3.87, 5.79)	4.65 (3.79, 5.70)	3.78 (3.05, 4.69)
	4th	11.44 (9.16, 14.29)	11.46 (9.12, 14.40)	8.76 (6.89, 11.12)

Results presented as odds ratios and 95% confidence intervals

^a^ Adjusted for age and sex

^b^ Adjusted for Model 1 + race/ethnicity

^c^ Adjusted for Model 2 + cardiovascular risk factors and comorbidities

Even stronger associations were observed in polynomial regression analyses of 2 or more financial toxicity components as the outcome of interest (when compared to reporting zero) (Table 4). In the fully adjusted model, participants in the 2nd, 3rd and 4th quartile had 2.46 (95% CI 1.79, 3.38), 7.73 (95% CI 5.77, 10.36), and 24.16 (95% CI 17.74, 32.90) higher adjusted relative prevalence of reporting any financial toxicity from healthcare compared with participants in the 1st quartile. Consistent qualitative trends were observed in analyses stratified by age.

**Table 4:** Associations between social determinants of healthquartiles and presence of 2 or more components of financial toxicity

	SDOH Aggregate Quartiles	Model 1^a^	Model 2^b^	Model 3^c^
**Overall**	*1st (Reference)*	*1.00*	*1.00*	*1.00*
	2nd	2.47 (1.83, 3.34)	2.48 (1.84, 3.36)	2.46 (1.79, 3.38)
	3rd	8.54 (6.50, 11.23)	8.87 (6.73, 11.68)	7.73 (5.77, 10.36)
	4th	27.00 (20.39, 35.75)	29.33 (22.03, 39.05)	24.16 (17.74, 32.90)
**< 65**	*1st (Reference)*	*1.00*	*1.00*	*1.00*
	2nd	2.69 (1.80, 4.01)	2.71 (1.81, 4.05)	3.06 (1.99, 4.72)
	3rd	7.02 (4.83, 10.22)	7.61 (5.23, 11.09)	8.05 (5.33, 12.16)
	4th	21.61 (14.91, 31.31)	24.71 (16.92, 36.09)	25.63 (16.74, 39.22)
**≥65**	*1st (Reference)*	*1.00*	*1.00*	*1.00*
	2nd	2.35 (1.50, 3.69)	2.35 (1.50, 3.70)	1.99 (1.25, 3.17)
	3rd	10.07 (6.59, 15.38)	9.67 (6.32, 14.81)	7.02 (4.50, 10.96)
	4th	35.87 (23.52, 54.72)	35.19 (22.96, 53.96)	24.39 (15.59, 38.16)

Results presented as relative prevalence ratios and 95% confidence intervals

^a^ Adjusted for age and sex

^b^ Adjusted for Model 1 + race/ethnicity

^c^ Adjusted for Model 2 + cardiovascular risk factors and comorbidities

### Sensitivity analyses

In a sensitivity analysis further stratified by income and not including income as part of the aggregate SDOH score, we observed that the burden of having ≥2 financial toxicity components was highest in participants with low income (Supplemental Fig. 1). In this analysis, trends in the prevalence of financial toxicity by SDOH quartiles remained roughly consistent with those observed in the overall study population, although the prevalence of financial toxicity now was also high among non-elderly low-income participants in the first SDOH quartile.

In a second sensitivity analysis restricting the aggregate SDOH score to 7 upstream SDOH factors, the qualitative trends were consistent with those observed using the 34-item SDOH score, although the associations were not as strong (Supplemental Table 3). Still, those in the most adverse SDOH group had 2.3-higher multivariable-adjusted odds of experiencing any financial toxicity compared with participants with zero adverse SDOH features. Analysis of individual SDOH revealed that being uninsured (OR = 2.65; 95% CI = 2.50, 2.81) was the strongest determinant of financial toxicity, followed by low income (OR = 1.26; 95% CI = 1.21, 1.32) (Supplementary Table 4). In additional analysis, we found a robust and consistent association between cumulative social disadvantage from “non-monetary” SDOH and financial toxicity (Supplementary Table 5A), albeit the observed association was relatively weaker in effect compared to the “monetary” SDOH effects model (Supplementary Table 5B).

Finally, using random forest regression, we found that “economic stability” was the SDOH domain that contributed the most to financial toxicity (43%), followed by “access to healthcare” (20%), “education” (15%), “community and social context” (14%), “food poverty” (7%), and “neighborhood” (2%) (Supplemental Fig. 2). This means that 57% of financial toxicity from healthcare can be attributed to SDOH beyond economic stability.

## Discussion

In this large, nationally representative study including 15,758 US patients with ASCVD, we found that an unfavorable SDOH profile, defined using an aggregate index combining 34 SDOH from 6 domains, was strongly and independently associated with subjective financial toxicity from healthcare. Individuals in the highest (i.e., most disadvantageous) SDOH quartile had a 68% prevalence of financial toxicity from healthcare and striking 8-fold higher multivariable-adjusted odds of experiencing financial toxicity than those in the most favorable group (SDOH quartile 1). Experiencing two or more components of financial toxicity was also very frequent among those in the most disadvantageous SDOH stratum (47% prevalence, and even higher [51% prevalence] among non-elderly participants from this subgroup). SDOH components other than economic stability significantly contributed to financial toxicity from healthcare, and consistent with this, the reported trends in financial toxicity from healthcare by SDOH quartiles persisted across strata of family income. In analyses stratified by age, similar trends were observed in both non-elderly and elderly patients, although financial toxicity was a much more frequent phenomenon in the non-elderly group. Sensitivity analyses restricted to 7 upstream SDOH yielded consistent qualitative trends.

The NHIS is the nation’s largest in-person household survey and represents a unique opportunity to study both SDOH and financial toxicity from healthcare, in a most granular manner and using a nationally representative sample of non-institutionalized US adults with ASCVD. To the best of our knowledge, this is the first population-based study in the US to examine this intersection in such a detailed manner among individuals with ASCVD — recognized by the American Heart Association as an important area of study in the field of cardiovascular health [21]. Our findings have important implications for policy moving forward.

Previous research from our group had shown that low income, a major SDOH, is independently associated with financial hardship from medical bills, inability to pay medical bills at all, as well as with potential consequences of these phenomena such as cost-related medication non-adherence and foregone care due to cost [8]. These associations were observed in US patients with ASCVD [4], as well as among those with cancer [22, 23], diabetes [24], chronic kidney disease [25], and worse cardiovascular risk profile overall [26]. In most of those analyses, a few other major SDOH such as education had been evaluated as potential predictors, however, were not significantly associated with difficulty paying medical bills and cost-related medication nonadherence after accounting for income [4, 5]. Our current analysis expands those prior efforts by evaluating a highly comprehensive index of cumulative SDOH disadvantage, and demonstrates that among patients with ASCVD, high social vulnerability is strongly associated with financial toxicity from healthcare, even after removing income and economic stability from the equation. Our findings suggest that besides income level and economic stability, which are major determinants of financial toxicity in patients with ASCVD, there are other SDOH characteristics the combined presence of which can put ASCVD patients at increased risk of financial toxicity. Specifically, our analyses of attributable risk suggest that healthcare system/access to care factors, education features, and community and social context characteristics also play relevant roles. These implications are also supported by findings from the sensitivity analyses (Supplementary Table 5), which suggests a strong and consistent association between non-monetary SDOH and financial toxicity, with nearly 3–4 fold higher likelihood of experiencing financial toxicity for individuals in the highest social disadvantage category (SDOH-Q4) for the non-monetary SDOH effects model (Supplementary Table 5A).

The cross-sectional nature of this analysis prevents us from drawing strong conclusions with regards to causality. In fact, reverse causation is a possibility: it is plausible that individuals with ASCVD who experience financial toxicity from healthcare may develop, later in time, downstream adverse SDOH such as high financial distress, psychological distress, or food insecurity, all of which were included in the comprehensive, 34 -item aggregate SDOH score. Nonetheless, in a sensitivity analysis that restricted the SDOH score to fewer, key features that are typically upstream, the associations were weaker but the reported trends remained present. Specifically, those in the most adverse SDOH stratum had 2.3 higher multivariable-adjusted odds of experiencing any financial toxicity than participants with zero adverse SDOH features.

Moving forward, our study together with previous research in this space have important implications. First, they provide further rationale to support policies aimed at improving the life conditions of those facing most adverse SDOH circumstances. A rich and robust body of literature has linked adverse SDOH with incident ASCVD and with adverse health outcomes among those with established ASCVD [27–29]. In this context, our study provides evidence of additional burdens faced by people with most adverse SDOH circumstances once they develop ASCVD, making an even stronger case for the need to protect these groups. As one of Healthy People 2030’s overarching 5 goals (“Create social, physical, and economic environments that promote attaining the full potential for health and well-being for all”) [30], future cardiovascular/public health research should focus on developing approaches that can tackle the shortcomings of adverse SDOH, as well as preventing and/or aiding with, or diminishing financial toxicity from healthcare in the population suffering from ASCVD. Importantly, the more upstream the interventions, the greater the impact: preventing ASCVD onset in SDOH vulnerable groups prevents both ASCVD and ASCVD-related financial toxicity altogether; and improving SDOH vulnerability can prevent not only ASCVD, but also many other conditions that disproportionately affect these groups [31].

Second, and while all patients with ASCVD in the US should be screened for and protected from financial toxicity from healthcare, and granted access to programs that help prevent and mitigate these phenomena, our analysis suggests that individuals with most adverse SDOH circumstances should be prioritized in those interventions, as their risk is highest. At the patient level, this would include improved access to financial navigators [32], enhanced training of clinicians to enhance their ability to engage in cost conversations with patients and prioritize less expensive therapeutic options when various, equally effective ones are available. For class I therapies that are out of reach but strongly indicated, further financial aid could be pursued [33]. At the system level, efforts should be made to enhance access to affordable care for ASCVD by socially vulnerable patients, while maximizing the quality and value of their care. Finally, large scale policy interventions such as “Medicaid expansion” have been shown to mitigate persistent financial barriers to healthcare, reduce foregone care and improve health outcomes, particularly among low income individuals and families in the US [34]. Ongoing efforts are needed to continue to expand the scope of such public welfare programs to address unfavorable SDOH such as lack of health insurance, food poverty, neighborhood disadvantage and homelessness, among others, and mitigate the risk of financial toxicity and related financial consequences of healthcare in socially vulnerable patients with ASCVD.

We observed roughly similar multivariable-adjusted associations between adverse SDOH and financial toxicity from healthcare among non-elderly and elderly participants. However, the proportion of non-elderly patients who developed financial toxicity was much higher than among elderly patients (45% vs 20%). Even though ASCVD is more frequent in the elderly, the absolute number of individuals experiencing any financial toxicity was also higher in the non-elderly group, and the same is true for inability to pay medical bills at all. These findings are consistent with our prior research [4, 7, 8], and the present study identifies non-elderly patients with ASCVD and adverse SDOH circumstances as a highly vulnerable group. The present study may also inform the development and validation of SDOH-based risk prediction scores that can help quantify the risk for healthcare-associated financial toxicity in patients with ASCVD as part of routine care.

### Study limitations

Besides the cross-sectional design, other limitations of the present analysis need to be.

acknowledged. First, the NHIS uses self-reported information, hence the potential for recall bias cannot be ruled out. Self-report of comorbidities may have resulted in misclassification, introducing residual confounding in the multivariable-adjusted models. Therefore, it is possible that the strong associations reported in our fully adjusted models may have been overestimated. Nonetheless, given the very comprehensive adjustment used in those analyses, the possibility of a fully spurious finding seems unlikely. Moreover, the need to adjust for comorbidities is debatable, as those (and the cost of their care) may be in the causal pathway through which adverse SDOH facilitate the development of financial toxicity among individuals with ASCVD.

Second, although we included a number of components of subjective financial toxicity from healthcare, objective financial toxicity was not evaluated. This information is not available in NHIS, but other surveys such as the Medical Expenditure Panel Survey (MEPS) may accommodate this research moving forward [7]. Along the same lines, we did not have information on other disease or healthcare-related factors such as total out-of-pocket costs spent in healthcare. Nevertheless, the areas of financial toxicity from healthcare that we studied have been previously validated and correlate well with studies that have included other features of healthcare and/or objective financial information [35].

Third, our aggregate score combined multiple SDOH items, all dichotomized and scored equally. While this is a rather simplistic modeling approach, it is important to stress that the purpose of our score was not to generate well-calibrated predictions at the individual level, but to identify subgroups accumulating the largest number of social vulnerability components. Scores that identify the number of adverse features are widely used in epidemiological research, health communication, and policy making. Examples include recent analyses of number of SDOH, fatal and nonfatal incident coronary heart disease in the Reasons for Geographic and Racial Differences in Stroke (REGARDS) study [28], to the American Heart Association’s “Life Simple Seven” [36], among many others. Nevertheless, we acknowledge that future risk prediction scores in this space might benefit from more complex statistical techniques. Future studies should pay greater attention to elucidating the possible mediators and moderators of the association between individual SDOH factors and financial toxicity, particularly for factors that are not fully understood with regards to their potential effects on financial toxicity, including the role of digital communication between the patient and provider, and other non-financial barriers to care [37].

Finally, we pooled 5 years of NHIS data to maximize power, precision, and generalizability. In this context, it is possible that secular changes could have occurred in the association between SDOH and financial toxicity during our study period. However, we would argue that a 5-year period is relatively short and that major changes are unlikely to happen.

## Conclusions

Among US patients with ASCVD, an unfavorable SDOH profile is strongly and independently associated with higher odds of experiencing subjective financial toxicity from healthcare. This association is consistent in non-elderly and elderly patients and is also present when analyses are restricted to non-economic SDOH domains and to most upstream SDOH features. Although the cross-sectional and observational nature of this study prevents from drawing causal conclusions, our analysis provides further evidence to support policies and interventions aimed at screening for prevalent financial toxicity and for high financial toxicity risk among socially vulnerable groups, and at improving their life conditions in the first place.

## Supplementary Information

**Additional file 1: Supplemental Table 1.** Social determinants of health components used, by domain. **Supplemental Table 2.** Components of financial toxicity from healthcare. **Supplemental Table 3.** Sensitivity analysis: Associations between 7 SDOH factors and any financial toxicity. **Supplemental Fig. 1a.** Prevalence of Any Financial Toxicity Among Adults < 65with and without ASCVD, by Family Income. **Supplemental Fig. 1b.** Prevalence of Any Financial Toxicity Among Adults **≥65** with and without ASCVD, by Family Income. **Supplemental Fig. 2.** Population Attributable Risk of Financial Toxicity by individual SDOH domains.

## Data Availability

The datasets generated and/or analyzed during the current study are obtainable in the National Health Interview Survey (NHIS) website, and they are publicly available at: https://www.cdc.gov/nchs/nhis/data-questionnaires-documentation.htm

## Abbreviations

ASCVD: Atherosclerotic cardiovascular disease
NHIS: National Health Interview Survey
SDOH: Social determinants of health
